#  Relapse of typhoid fever following delayed response to meropenem: A case report and review of previously published cases indicating limited clinical efficacy of meropenem for the treatment of typhoid fever

**DOI:** 10.3205/000267

**Published:** 2019-01-07

**Authors:** Christian G. Blumentrath, Gernot Müller, Dieter Teichmann, Jens Tiesmeier, Jasmina Petridou

**Affiliations:** 1 Clinic for Cardiology, Angiology and Intensive Care Medicine, Klinikum Lippe Detmold, Germany; 2 Department of Infectious Diseases and Tropical Medicine, Städtisches Klinikum Dresden, Germany; 3 Institute of Anaesthesiology, Intensive Care and Emergency Medicine, General Hospital Lübbecke-Rahden, Germany; 4 Institute of Medical Microbiology, University Hospital Minden, Germany

**Keywords:** tolerance, persistence, salmonella, treatment failure, resistance

## Abstract

In times of emerging multi-drug resistance among Gram-negative bacteria (including *Salmonella enterica*, Serovar Typhi), we observed relapse of typhoid fever following delayed response to treatment with meropenem, suggestive for limited clinical efficacy of the drug. Three previously published cases supported our suspicion. Within this context, we discuss the case details with a focus on potential explanations for insufficient clinical response to meropenem (e.g. limited intracellular penetration, phenomena of tolerance and persistence). Meropenem is a last-resort antimicrobial agent for the treatment of multi-drug resistant Gram-negative infections. Reliable clinical data evaluating the efficacy of meropenem for the treatment of typhoid fever are urgently needed. Future clinical studies evaluating typhoid fever outcome should also investigate the impact of (i) intracellular penetration of antibiotics, and (ii) tolerance and persistence on outcome.

## Zusammenfassung

In Zeiten zunehmender Multiresistenzen bei Gramnegativen Erregern (*Salmonella enterica*, Serovar Typhi eingeschlossen) beobachteten wir einen Rückfall nach verzögertem Ansprechen eines Typhus-Patienten auf die Behandlung mit Meropenem. Diese Beobachtung legte den Verdacht nahe, die Wirksamkeit des Antibiotikums könne bei Typhus-Patienten eingeschränkt sein. Drei bereits veröffentlichte Fälle bestätigten diesen Verdacht. Wir diskutieren die Details des Falles, legen den Schwerpunkt der Diskussion aber auf mögliche Erklärungen für ein vermindertes Ansprechen der vier Typhus-Patienten auf Meropenem. Meropenem ist ein Reserve-Antibiotikum zur Behandlung multiresistenter, Gramnegativer Infektionen. Verlässliche Daten zur Wirksamkeit von Meropenem bei Typhus-Patienten werden dringend benötigt. Zukünftige Studien zur Wirksamkeit von Antibiotika zur Behandlung des Typhus sollten auch (i) die Bedeutung des intrazellulären Eindringens der Medikamente und (ii) den Einfluss von Toleranz und Persistenz auf die Wirksamkeit der Therapie untersuchen.

## Introduction

While bacterial infections such as typhoid fever had formerly lost much of their terror due to improved sanitation, appropriate antibiotic therapy, consequent infection control, and surveillance measures of cases in industrialized countries [1], [2], the disease remains a major public health problem in resource-limited, endemic countries [2]. The Robert Koch Institute reports approximately 50 cases of typhoid fever in Germany annually, most of them acquired in India and other endemic areas [3]. Hence, in numerous medical institutions in non-endemic countries, experience concerning diagnosis and treatment of typhoid fever is limited. In Germany, suspicion or confirmation of disease, as well as death due to *Salmonella enterica* Serovar Typhi (S. Typhi) are notifiable [3].

Following exposure (faecal-oral transmission: contact to typhoid fever patients or chronic carriers, or ingestion of contaminated food or beverages), it takes 10–14 days (range: 3–60 days, depending on the number of bacteria ingested), until the first symptoms occur [2], [3], [4].

The initial symptoms are unspecific: general malaise, stepladder-fever (over 3–4 days), headache, sore throat, dry cough, muscle and joint pain, constipation, or diarrhoea (approximately 48% of the patients display diarrhoea on admission) [2].

Clinical examination may reveal: relative or absolute bradycardia, high-grade fever and rose spots (rather rare) [2], [4]. Laboratory examination may corroborate the initial suspicion: normal white blood count, mild thrombocytopenia, eosinopenia or aneosinophilia, moderately elevated C-reactive protein (CRP) and lactate-dehydrogenase (LDH) [2], [4]. During the second week of infection, alanine- (ALT) and aspartate-aminotransferase (AST) increase. A 2–3 fold elevation of liver enzyme levels (AST and ALT) is a common characteristic of the disease [2], [4], [5]. Blood cultures are the gold standard for the detection of S. Typhi [2], [3], [4]. Sensitivity declines over time: in the first week to 90%; in the second week to 75%; in the third week to 60%; in the fourth week to 25% [4]. Bone marrow culture may reveal bacteria in late disease if blood cultures remain negative [4]. Sensitivity of stool culture is poor (approximately 40%) – polymerase chain reaction (PCR) improves sensitivity of blood, stool, and urine examination [6]. Serology and Widal reaction are unspecific [4].

Early and appropriate antibiotic treatment significantly reduces complication rate, rate of chronic carriers, and mortality (up to 30% in the pre-antibiotic era versus virtually no deaths in returning travellers) [4], [7].

Multi-drug resistance among Gram-negative bacterial infections (including S. Typhi) is alarmingly increasing [8], [9]. Although multi-drug resistance rates vary largely among different geographic regions (e.g. up to 70% in some hospitals in India vs. less than 7% in European countries), infections due to these bacteria provide a challenge to modern medicine [8], [9]. Global warming, increased migration, tourism and international trading, as well as public impoverishment (of marginalised groups) inevitably result in the globalisation of infectious diseases, and facilitate their spreading [8], [10]. As observed in our case, ciprofloxacin-resistant S. Typhi strains are associated with a growing number of complications [11], [12]. Therefore, physicians should be aware of red flag features (Table 1 (Tab. 1)) and the following factors indicating complications:

- infections with multi-drug resistant strains [2], [4], [10], [13];
- state of immunosuppression, e.g. HIV, malnutrition [2], [4], [10], [13], [14];
- structural and functional abnormalities, e.g. malignant tumours [15], haemoglobinopathies (Sickle cell disease) [16], [17], cysts [18], neurologic disorders [19];
- infants/young children, elderly patients [2], [4], [10], [13];
- patients with limited access to proper health care, e.g. patients from remote areas or low-income countries, patients affected by poverty [2], [4], [13];
- delay in diagnosis and treatment [2], [4], [13];
- inappropriate antibiotic treatment, e.g. short-course therapy, not according to sensitivity testing [2], [4], [10], [13];
- inoculation of a huge number of bacteria [2], [4], [13];
- strain-related virulence factors [2], [4], [10], [13].

Rarely, patients may develop late onset and persisting complications:

- relapse (14 days up to 3 months following treatment) [4];
- psychiatric disorders [20];
- neurologic disorders [21], [22], [23];
- ophthalmologic disorders [24];
- intracranial abscess (47 years following typhoid fever) [25];
- atrophic rhinitis [26].

Here, we are analysing a case of relapse following treatment of typhoid fever using meropenem. The case illustrates the diagnostic and therapeutic difficulties which arise from the above-mentioned problems. Furthermore, it is the fourth case questioning the efficacy of meropenem for the treatment of typhoid fever.

## Case description

A previously healthy, Caucasian, 18-year-old man presented at our Department of Emergency Medicine for watery diarrhoea, high-grade fever, and severe malaise. Stool samples performed by the family physician had been negative, including testing for *Salmonella* species (spp.). Five days after returning from travelling to various countries, e.g. India and Nepal, he developed fever, chills, cough, sore throat, and headaches, which lasted for 3 days before diarrhoea started. The total duration of the disease on admission was 7 days.

### Travel destinations

- day 1: Germany
- day 2–4: Kingdom of Bahrain
- day 4–8: United Arab Emirates
  (Dubai: day 4–6; Abu Dhabi: day 6–8)
- day 8–11: Kathmandu, Nepal
- day 11–13: Delhi, India
- day 13–16: Kuala Lumpur, Malaysia
- day 16–18: Singapore
- day 19–23: Melbourne, Australia
- day 24–26: Taipei, Taiwan
- day 26–28: Manila, Philippines
- day 28–29: Kuwait City, Kuwait

During his travels, he experienced gastroenteritis while he was in Delhi (oral antibiotic therapy and electrolyte solution resulted in cure after 3 days), and multiple mosquito bites in malaria-endemic countries. He denied tick bites and animal contact of all kinds. Although he had received pre-travel medical advice, he did not respect alimentary precautions (he preferred vegetables and salad in local restaurants), and had refused malaria prophylaxis due to fear of side effects. The patient did not receive any vaccination against cholera or typhoid fever.

Apart from signs of exsiccosis, a thorough physical examination was unremarkable. The patient was fully conscious, had a relative bradycardia (95/min), hypotension (95/60 mmHg), high-grade fever (39°C), and normal oxygen saturation. Electrocardiogram was normal and abdominal ultrasound was consistent with diagnosis of gastroenteritis. A differential blood count demonstrated a normal white blood count, aneosinophilia and discrete thrombocytopenia (126/µl, normal: 140–200/µl). Laboratory examination revealed an elevated CRP level (93 mg/dl; normal: <5 mg/dl) and slightly elevated ALT (48 U/l, normal: <40 U/l), AST (59 U/l, normal: <41 U/l) and LDH (461 U/l, normal: <250 U/l). Creatinine levels, blood gas and urine analysis were normal. Malaria was ruled out using thick smears and rapid testing. Blood, urine, and stool cultures were performed. The latter two showed no growth.

Due to suspected typhoid fever, we started intravenous ceftriaxone (2 g once daily), fluid supplementation (2.500 ml per day), and oral antipyretics (750 mg metamizole four times daily). Liver enzymes increased to ALT 97 U/l and AST 83 U/l on day 8 after disease onset; to ALT 196 U/l and AST 165 U/l on day 9 after disease onset. Diarrhoea subsided to 15–20 times per day; fever subsided as well. On day 11 after disease onset, blood cultures revealed Gram-negative bacteria. By the time, the patient’s condition had not improved. We suspected a Gram-negative sepsis and changed the antibiotic regime to intravenous meropenem, 1 g three times per day. One day later, *Salmonella enterica* Serovar Typhi was identified (susceptibility testing: Table 2 (Tab. 2)), and the therapy was continued with meropenem. Liver enzymes peaked on day 12 after the onset of initial symptoms: LDH 756 U/l, ALT 544 U/l, AST 263 U/l, alkaline phosphatase (AP) 168 U/l (normal: 55–149 U/l), and Gamma-GT 196 U/l (normal: <60 U/l). Bilirubin remained normal; abdominal ultrasound displayed mild hepato- and splenomegaly. We ruled out hepatitis A/B/C/D/E (serologic tests), entero-haemorrhagic *Escherichia coli*, and amoebic liver disease (stool samples), and continued the treatment regime. On day 16 after disease onset (day 9 of antibiotic therapy), the patient’s condition improved, both his body temperature and his liver enzymes decreased. On day 14 of treatment (4 days ceftriaxone; 10 days meropenem), the patient had fully recovered (including complete normalization of laboratory parameters). One week after treatment, 3 stool samples (obtained on 3 different days) were negative for S. Typhi.

During a family visit in Dresden 14 days after completion of initial treatment, the patient was hospitalised again (Department of Infectious Diseases and Tropical Medicine, Städtisches Klinikum Dresden) for high-grade fever, crampy abdominal pain, and watery diarrhoea. Abdominal ultrasound revealed extended mesenteric lymph nodes. The colleagues ruled out schistosomiasis and HIV (serology), as well as helminthic infections, other parasites, and *Clostridium difficile* (stool analyses). Blood cultures revealed S. Typhi (susceptibility testing: Table 2 (Tab. 2)). Urine analysis, performed because of dysuria and pollakiuria, yielded a urinary tract infection (UTI) due to *Escherichia coli* (4-MRGN (German Classification of Gram-negative bacteria indicating resistance to 4 clinically relevant groups of bactericidal antibiotics: cephalosporine and acylureidopenicilline antibiotics, carbapenems and fluoroquinolones [7]), OXA-48 positive; Colony forming units: 10^6^/ml).

Consequently, the colleagues administered a combination antibiotic therapy according to susceptibility testing using intravenous ceftriaxone (2 g daily dose maintained for 28 days to address relapse) and oral sulfamethoxazole/trimethoprim (800/160 mg daily dose, maintained for 10 days to address the UTI). The patient fully recovered. Again, 3 stool samples following treatment were negative for S. Typhi. The patient has been free of relapse for 9 months.

## Discussion

The case report recalls the importance of individualized pre-travel medical advice, illustrates diagnostics of fever in a returning traveller, and demonstrates that increasing multi-drug resistance among Gram-negative bacteria impairs treatment and outcome of typhoid fever. Notably, delayed response to treatment with meropenem followed by relapse challenges the efficacy of a last-resort antimicrobial agent.

Overall, pre-travel medical advice of our patient was poor (no alimentary precautions, no vaccination, no malaria prophylaxis). Individualised pre-travel medical advice including vaccination against typhoid fever might have prevented the infection [27], [28]. However, protection following immunisation is limited (75%), and there is an urgent need for improved typhoid fever vaccination [4].

The patient’s history as well as clinical and laboratory findings matched typhoid fever (compare: introduction section) [4]. Additionally, important differential diagnoses were ruled out by clinical and laboratory examinations [29]. Therefore, suspicion of typhoid fever and immediate administration of ceftriaxone were justified.

The decision to switch antimicrobial treatment to meropenem on day 5 of treatment was based on case deterioration and the increasing prevalence of MDR and extended spectrum of ß-lactamase producing (ESBL) Gram-negative bacteria (including* Salmonella* spp.) in countries which our patient had travelled to (e.g. India and Nepal) [8], [9]. However, some reasons argue against this switch. First, the expected fever clearance time of typhoid fever is approximately 7 days from treatment initiation (range: 3–12 days), depending on the antibiotic used [2], [4], [30], [13], [31], [32], [33]. Second, the patient did not match sepsis criteria by the time of the regime change [34]. As meropenem is a last-resort antimicrobial agent for the treatment of multidrug-resistant Gram-negative infections [8], it would have been reasonable to wait for the results of susceptibility testing. Once the results were available (Table 2 (Tab. 2)), return to ceftriaxone was indicated [3], [8].

Although reliable clinical data supporting the use of meropenem for the treatment of typhoid fever is limited to* in vitro* susceptibility testing and a few case reports [9], [35], [36], we completed treatment using meropenem. Indeed, the drug did not meet the expectations. A literature search revealed three other case reports which also questioned the clinical efficacy of meropenem [35], [36], [37].

The isolates of all four cases (throughout the manuscript, all four cases refer to: this report, Kleine et al. [35], Godbole et al. [36], and Lukácová et al. [37]) did not adequately respond to meropenem monotherapy, although the isolates were fully susceptible (Table 2 (Tab. 2)) [35], [36], [37]. All four isolates demonstrated ciprofloxacin resistance and two isolates were resistant to ceftriaxone as well (Table 2 (Tab. 2)). None of the patients displayed any underlying conditions (e.g. immunosuppression, adherence of bacteria to artificial material, abscesses) which might explain the inadequate response [35], [36], [37].

Godbole et al. proposed that limited intracellular penetration of meropenem may be responsible for treatment failures [36]. The observation that ciprofloxacin and azithromycin (both accumulate intracellularly, the latter even in lysosomes [38], [39]) were particularly effective against susceptible S. Typhi strains, stresses the importance of an intracellular action of the antimicrobial agent [36]. However, excellent response to treatment with meropenem was reported, too [40]. Additionally, limited intracellular penetration (more precisely, lack of intracellular accumulation) is the case for all ß-lactam-antibiotics, including amoxicillin, ampicillin, and ceftriaxone [38], [39], which have been successfully used to treat typhoid fever [2], [4], [30].

Therefore, the phenomena of tolerance and persistence (as defined by Kerster and Fortune [41]) provide alternative explanations [42]. Due to slow growth and dormancy, tolerant bacteria temporarily survive exposure to concentrations of antimicrobial agents, which are normally lethal [42]. If only a small bacterial subpopulation demonstrates the same capability, this is termed persistence (not to be confused: an infection which is not effectively cleared in the host is also referred to as persistent) [42]. The phenomena can be inherited (e.g. tolerance mutations in a toxin-antitoxin module), or acquired (e.g. induced by antibiotics) [42], [43]. Treatment failure due to tolerance and persistence occurs, although the Minimal Inhibitory Concentration (MIC) of the antibiotic used is well below the breakpoint (matches all four cases) [42], [43]. This implicates that survival is not related to any resistance phenotype [42], [43]. Currently, there are two options to detect tolerance and persistence: determination of the minimum duration to kill 99% (MDK_99_ to detect tolerance) and 99.99% (MDK_99.99_ to detect persistence) of a given bacterial population [42]; another, simpler option is the Tolerance Diffusion Test (TDtest) as provided by Grefen et al. [43]. In addition, some tolerance mutations can be detected using molecular techniques (e.g. detection of a mutation in the vapBC toxin-antitoxin module) [42], [43]. Unfortunately, none of these analyses were performed for any of the four cases.

However, S. Typhi meets the prerequisites of tolerance and persistence [42]:

1. phenotypic variation in host tissues, which lead to delayed eradication [44],
2. formation of antibiotic-tolerant subpopulations [45],
3. formation of nonreplicating persisters [46].

We believe that the phenomena of tolerance and persistence are most appropriate to explain the limited response to meropenem in all four cases, as well as the relapse in our case.

Survival due to tolerance and/or persistence is temporary [42], [43]. Antibiotics with a short half-life time (e.g. amoxicillin, meropenem) may therefore be more likely to help bacteria evolve tolerance (which may reach 100%), compared to antibiotics with a longer half-life time (e.g. ceftriaxone, azithromycin) [42], [43]. Lukácová et al. obtained no response to several bactericidal antibiotics administered according to susceptibility testing, including meropenem [37] – perhaps because switching between bactericidal antibiotics is not suitable for overcoming tolerance and persistence [42]. Kleine et al. reported case-deterioration although they doubled the dosages of meropenem [35]. In contrast to proper resistance, which can be overcome by increasing dosages, such action does not adequately address tolerance and persistence [42], [43]. Response of the case (reported by Kleine et al. [35]) following the addition of fosfomycin on day 19 of meropenem monotherapy may be coincidental – the phenomena respond to prolonged treatment durations [42] –, or a direct effect of combination, because combination antimicrobial therapy may overcome tolerance and persistence. The efficacy of bacteriostatic antibiotics is not affected by the phenomena [42]. Accordingly, two cases responded to bacteriostatic antibiotics (chloramphenicol: Lukácová et al. [37], and azithromycin: Godbole et al. [36]) following insufficient treatment with bactericidal antibiotics, e.g. meropenem) [36], [37]. For the case reported by Godbole et al., one may also assume an effect of combination therapy (4 days meropenem alone, 10 days meropenem and azithromycin in combination) [36].

In fact, one study indicates that the combination of ceftriaxone and azithromycin reduced bacteria- and fever-clearance times when compared to monotherapy [47]. Therefore, if treatment with meropenem is unavoidable, we agree with Kleine et al. and Godbole et al. that meropenem should be combined with an antimicrobial agent [35], [36] which preferably provides an intracellular mode of action [36] and a long half-life time – at least for severe typhoid fever cases [35], [36].

Our patient relapsed 14 days after completion of treatment (relapse usually occurs within up to six weeks after treatment [4]). If meropenem is as effective as ceftriaxone, our patient displayed only one risk factor for relapse (isolated ciprofloxacin resistance) out of seven risk factors described in medical literature:

1. the drug chosen for treatment (cephalosporines other than ceftriaxone > ceftriaxone > ciprofloxacin > azithromycin);
2. duration of treatment;
3. constipation on admission;
4. fever within 14 days of admission;
5. HIV co-infection;
6. infection with multi-drug resistant/ciprofloxacin resistant strains;
7. anatomical and structural abnormalities (e.g. schistosomiasis eggs, gallstones) [2], [4], [11], [12], [13], [30], [31], [32], [33].

We believe that the patient relapsed due to reactivation of dormant bacteria which disseminated from mesenteric lymph nodes, a mechanism suggested by Griffin et al. [48]. It is quite possible that meropenem does not adequately target intracellular, dormant bacteria [36], [42]. Increased treatment durations reduced relapse rates of typhoid fever patients [33]. The fact that such action is suitable for overcoming tolerance and persistence [42] supports our assumption. Furthermore:

1. on admission for relapse, mesenteric lymph nodes of our patient were markedly distended;
2. three negative stool samples indicate that the hepatobiliary system was probably not the source of relapse;
3. clinical cure and complete normalisation of laboratory parameters (including normalisation of CRP) made relapse from abscesses unlikely.

In the absence of recommendations for the treatment of relapse (in general, relapse occurs two to six weeks following initial treatment [4]), we performed a long-term treatment (ceftriaxone for 28 days). Others preferred even longer treatment durations (e.g. 60 days) [5]. Azithromycin would most likely have been a better option [2], [4], [13], [30], [31], [32], [33], but unfortunately, our susceptibility testing (Table 2 (Tab. 2)) did not include the drug.

With the urinary tract infection that resulted from a multi-drug resistant *Escherichia coli* (4-MRGN, OXA-48 positive), the case came full circle. Plasmid-encoded resistance genes are highly transmissible among Gram-negative bacteria. Since regions where multi-drug resistant Gram-negative infections frequently occur (e.g. India) largely overlap with regions where typhoid fever is endemic, we might soon be faced with the challenge of untreatable typhoid fever [8], [9].

## Conclusions

The case report illustrates that emerging multi-drug resistant typhoid fever is a threat to people residing in or travelling to endemic countries. Our analysis stresses the need for reliable clinical data evaluating the efficacy of carbapenems (e.g. meropenem) for the treatment of typhoid fever, and emphasizes the importance to further investigate the impact of tolerance and persistence on treatment and outcome (e.g. correlate the results of TD tests with clinical outcome). New strategies for infection prevention (e.g. new and better vaccines) and new treatment options (e.g. new antimicrobial agents) are urgently needed.

## Notes

### Competing interests

The authors declare that they have no competing interests.

### Financial disclosure

The authors received no funding for this analysis.

### Acknowledgements

The authors thank their colleagues (physicians and nurses, laboratory, radiology, and cleaning staff) for their great support in daily practise. The authors also thank Susan Rüther, teacher for English language and history at Gymnasium Aspel in Rees, Germany, for English language editing. CGB thanks his former colleagues from the General Hospital Lübbecke-Rahden (the case was diagnosed and followed-up in this hospital) for their great support during collaboration.

### Authors’ contributions

CGB conceived the idea for this article, performed literature search and drafted the first version of the manuscript. CGB, GM, and TD guided diagnosis, treatment, and follow-up of the patient. All authors reviewed the results of literature search and contributed to the final version of the manuscript. All authors read and approved the final version of the article.

## Figures and Tables

**Table 1:**
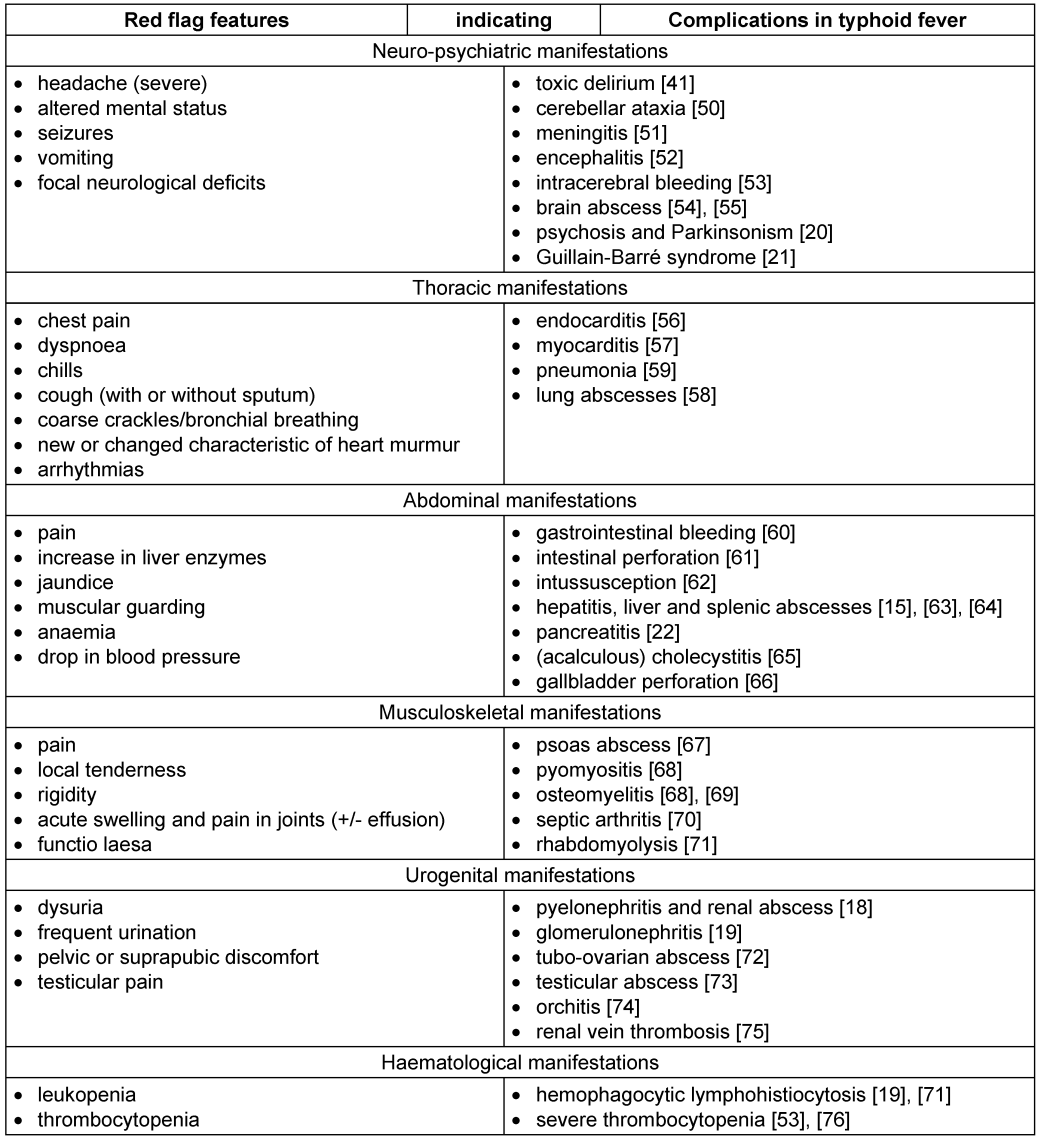
Red flag features with corresponding complications (modified after Upadhyay R et al. [4])

**Table 2:**
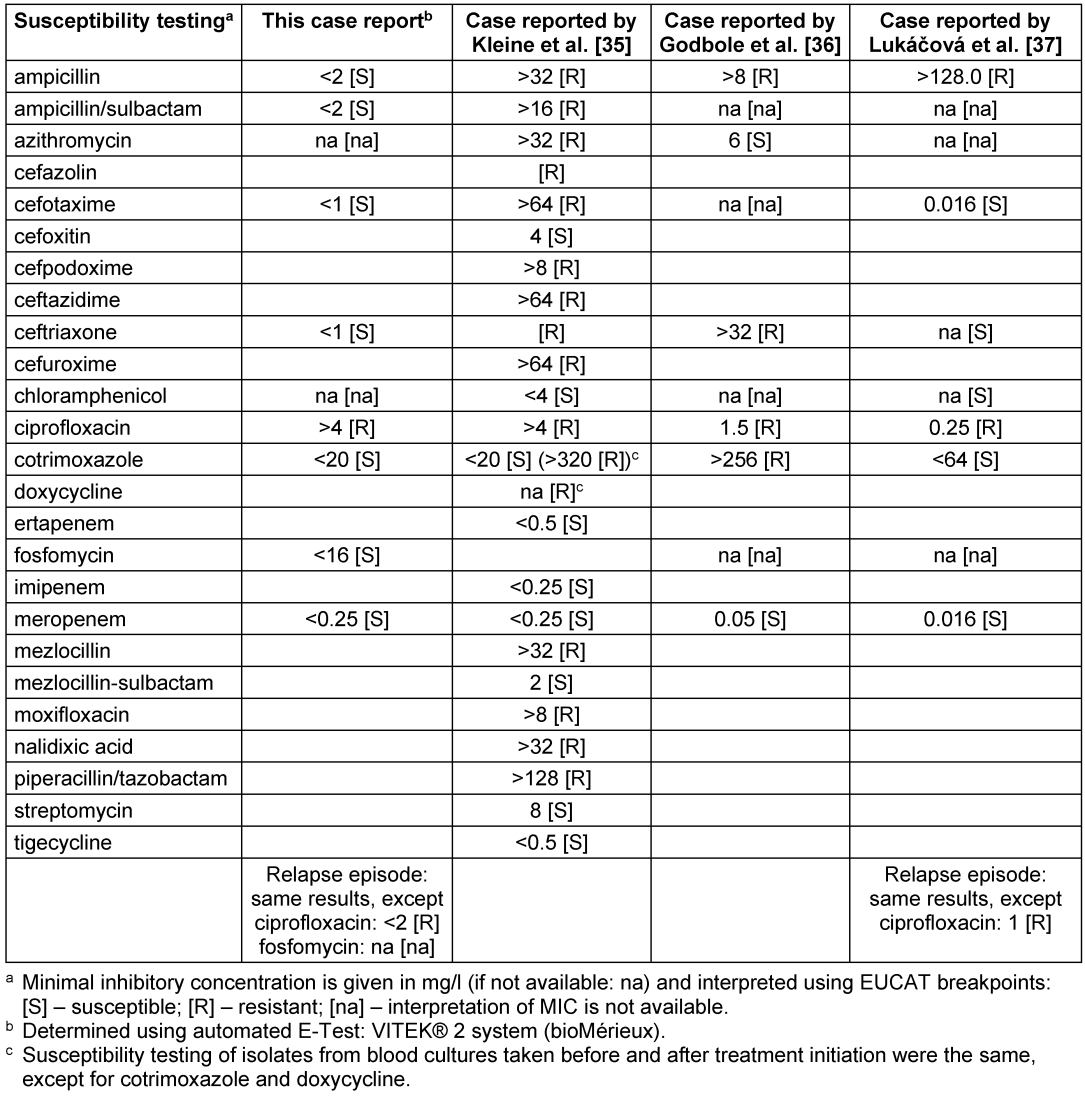
Susceptibility testing of this report and the three previously published cases indicating limited clinical efficacy of meropenem for the treatment of typhoid fever
